# Prevalence and potential risk factors of hypokalemia in pediatric patients with diabetic ketoacidosis

**DOI:** 10.1186/1687-9856-2015-S1-P8

**Published:** 2015-04-28

**Authors:** Lai Ka Lee, Chun Hung Ko, Ching Yin Lee, Ching Ngar Hung, Nai Chung Fong, Wai Lim Yiu

**Affiliations:** 1 Department of Pediatrics, The Prince of Wales Hospital, Hong Kong, China; 2 Department of Pediatrics and Adolescent Medicine, Caritas Medical Center, Hong Kong, China; 3 Department of Health, Hong Kong, China; 4 Department of Paediatrics and Adolescent Medicine, Princess Margaret Hospital, Hong Kong, China

## Aims

To examine the local prevalence of hypokalemia in patients with diabetic ketoacidosis (DKA), both at presentation and during treatment, and to investigate the potential risk factors leading to significant hypokalemia during treatment of DKA.

## Methods

Retrospective review of 114 consecutive patient-episodes. Univariate analyses were performed to study any difference in mean between the group with nadir of potassium (Kn) >= 3.0mmol/L from group with Kn < 3.0mmol/L for predictors concerning patients’ demographics, the baseline characteristics, the therapies for DKA (including average insulin infusion rate before Kn), and the pace of recovery from DKA. Predictors deemed statistical significant in univariate analyses were subjected to multivariate analysis.

## Results

The period prevalence of hypokalemia at presentation and during treatment of DKA were 13.8% and 92.5% respectively. Univariate analysis showed patients who were younger, with lower mean body weight, lower mean plasma bicarbonate at presentation, lower mean serum potassium level at presentation, higher urine output per unit body weight (in the first 24 hours of admission), higher amount of potassium supplement given before Kn, shorter time lag of starting potassium supplements (as reference to time of start of insulin) and longer duration of metabolic acidosis were independently associated with risk of developing Kn < 3.0mmol/L. Multivariate analysis showed that duration of metabolic acidosis was the sole risk factor for having Kn < 3.0mmol/L.

## Conclusions

In our cohort, the longer duration of metabolic acidosis predicts significant hypokalemia during DKA treatment, which could have represented a persistent accumulation of free fatty acid and an on-going stimulus for aldosterone secretion, hence kaliuresis-related hypokalemia [1-7]. Therefore, in patients with slow resolution of metabolic acidosis, the measurement of the urinary potassium might allow for better estimation of potassium requirement during DKA treatment, such that significant hypokalemia could be minimized. In our data, the average insulin infusion rate was not associated with statistically increased risk of significant hypokalemia, therefore, the strategy of lowering insulin infusion rate in patients with significant hypokalemia during DKA treatment should require further evaluation.
